# Metformin-Loaded Chitosan Hydrogels Suppress Bladder Tumor Growth in an Orthotopic Mouse Model via Intravesical Administration

**DOI:** 10.3390/molecules28186720

**Published:** 2023-09-20

**Authors:** Xingjian Zhang, Xin Hu, Yijun Xie, Lejing Xie, Xiangyi Chen, Mei Peng, Duo Li, Jun Deng, Di Xiao, Xiaoping Yang

**Affiliations:** 1 Key Laboratory of Study and Discovery of Small Targeted Molecules of Hunan Province, Key Laboratory of Chemical Biology & Traditional Chinese Medicine Research of Ministry of Education, Department of Pharmacy, School of Medicine, Hunan Normal University, Changsha 410205, China; 202130193046@hunnu.edu.cn (X.Z.); xinhu@hunnu.edu.cn (X.H.); 202320193580@hunnu.edu.cn (Y.X.); 202030192021@hunnu.edu.cn (L.X.); xiangyichen@hunnu.edu.cn (X.C.); meipeng@csu.edu.cn (M.P.); liduo@hunnu.edu.cn (D.L.); 18390839921@163.com (J.D.); 2 Key Laboratory of Protein Chemistry and Developmental Biology of Fish of Ministry of Education, Hunan Normal University, Changsha 410205, China

**Keywords:** chitosan hydrogels, bladder cancer, metformin, intravesical administration, orthotopic model

## Abstract

Our previous study found that the intravesical perfusion of metformin has excellent inhibitory effects against bladder cancer (BC). However, this administration route allows the drug to be diluted and excreted in urine. Therefore, increasing the adhesion of metformin to the bladder mucosal layer may prolong the retention time and increase the pharmacological activity. It is well known that chitosan (Cs) has a strong adhesion to the bladder mucosal layer. Thus, this study established a novel formulation of metformin to enhance its antitumor activity by extending its retention time. In this research, we prepared Cs freeze-dried powder and investigated the effect of metformin-loaded chitosan hydrogels (MLCH) in vitro and in vivo. The results showed that MLCH had a strong inhibitory effect against proliferation and colony formation in vitro. The reduction in BC weight and the expression of tumor biomarkers in orthotopic mice showed the robust antitumor activity of MLCH via intravesical administration in vivo. The non-toxic profile of MLCH was observed as well, using histological examinations. Mechanistically, MLCH showed stronger functional activation of the AMPKα/mTOR signaling pathway compared with metformin alone. These findings aim to make this novel formulation an efficient candidate for managing BC via intravesical administration.

## 1. Introduction

Bladder cancer (BC) is the most common malignancy of the urinary system [1]. At present, the most common treatment approach for BC is still transurethral resections of the bladder tumor (TURBT) and intravesical drug delivery (IDD) of Bacillus Calmette–Guérin (BCG) [2]. However, these treatments have a high recurrence rate and a severe risk of progression remains. Recently, PD-1/PD-L1 inhibitors and FGFR inhibitors were approved for BC but potential side effects and the low response ratio of these drugs have restricted their clinical application [3]. Thus, novel therapeutic approaches are urgently required.

Metformin (Met, Figure S1) has been a first-line oral hypoglycemic agent for the treatment of type 2 diabetes (T2D) for decades and its potential association with decreased cancer risk and improved cancer prognosis has stimulated increasing attention [4]. However, according to the results of multiple clinical trials, metformin has not been effective in inhibiting the proliferation and growth of tumor cells if taken orally [5,6,7]. It has been speculated that the low clinical anticancer responses of metformin are because of the circulating concentration in the body being low following conventional drug delivery, failing to achieve a sufficient dose to kill the tumor cells. The unique physiological features of the bladder allow the intravesical administration of metformin, which can enhance the anti-tumor concentration locally [8]. However, intravesical administration has inherent limitations since the delivered drugs may be diluted and discharged easily in the urine [9]. Consequently, to attain an effective concentration, repeated intubation is acquired, which puts patients at increased risk of infection and inconvenience. Reducing the dose and increasing the retention time on the bladder surface remains a challenge.

For the sake of solving the problems above-mentioned, according to the negatively charged properties of the bladder mucosa, we sought for a positively charged drug-carrying material, namely chitosan (Cs), which is easily adsorbed by bladder mucosa according to charge adsorption to increase the drug retention time. Studies have shown that chitosan is a nature-derived mucopolysaccharide primarily attained from the deacetylation of chitin [10,11,12]. In recent years, benefiting from its nanometric size, biodegradability, virulence, and biocompatibility, Cs has been used to deliver various different therapeutic agents, including peptides, small molecules, vaccines, DNA, and drugs [13,14]. Thus, developing novel formulations using Cs as a carrier for treating BC has significant potential in anti-BC drug development. The present study prepared metformin-loaded Cs hydrogels (MLCH) using freeze-drying to enhance the bioavailability of the metformin when intravesically administered. Moreover, a cell culture model was used to determine the optimized formulation. An orthotropic animal bladder tumor model, which simulates the occurrence and development of human BC [15], was used to evaluate the efficiency of this newly prepared MLCH formulation. Significantly, based on previous explorations of the molecular mechanism of metformin [16,17], the functional activation of the AMPKα/mTOR signaling pathway was determined using both in vitro and in vivo samples. The aim of this study is to pave the way for exploring the development of safe and highly efficient formulations of Met for clinical use in the treatment of BC.

## 2. Results

### 2.1. Freeze-Drying Improves the Microstructure of Cs and Induces the Formation of Colloidal Hydrogels

**Figure 1:**
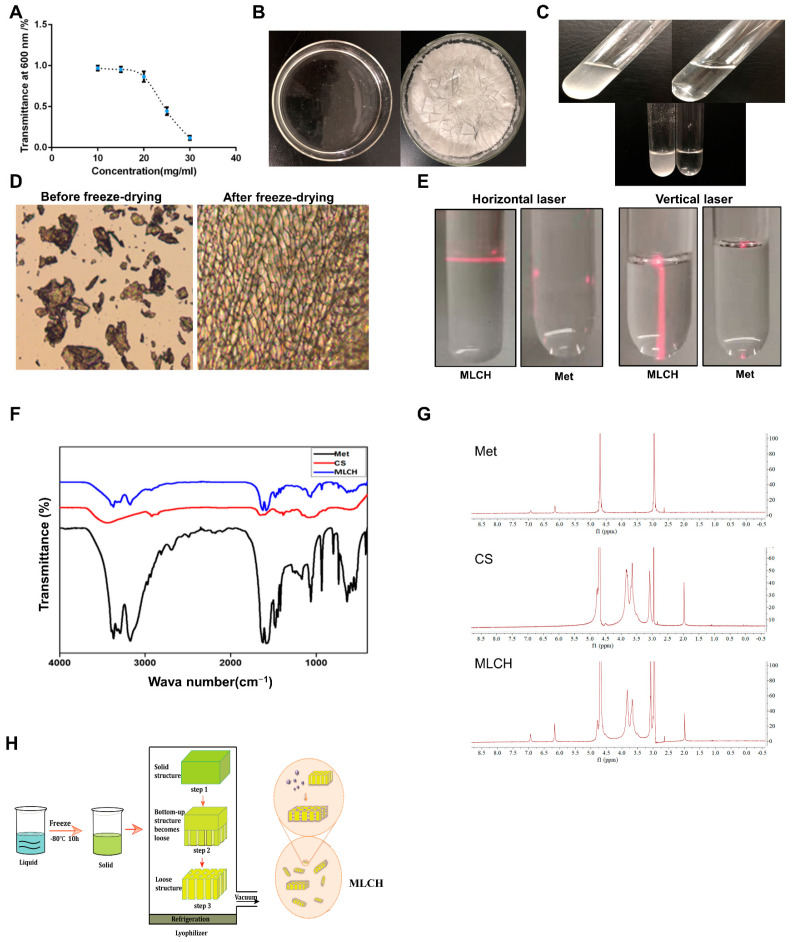
Physical characteristics of Cs hydrogels. (**A**) Concentration-dependent transmittance changes in the Cs hydrochloride solution at a wavelength of 600 nm. Data are presented as the mean ± standard deviation of three repetitions. (**B**) After placing it in a vacuum at low temperature, the clear solution (left) of regular Cs powder dissolved in 1% hydrochloric acid (*v*/*v*) became a uniform of lyophilized Cs powder (right). (**C**) Lyophilized Cs powder could be dissolved in Met physiological saline to form even MLCH (right) whereas regular Cs powder could not (left). (**D**) Micro-structural differences between the regular and lyophilized powder. The images were taken using an inverted microscope. (**E**) Results of the Tyndall effect assay on MLCH and Met. (**F**) Infrared spectra of MLCH, Met, and Cs hydrogels. (**G**) ^1^H NMR spectra of MLCH, Met, and Cs hydrogels. The solvent used for ^1^H NMR spectra is D_2_O. (**H**) The prepared Cs lyophilized powder showed a loose and porous three dimensional network structure so it has a high metformin loading capacity. MLCH, metformin-loaded Cs hydrogel; Met, metformin.

The transmittance measured by UV–vis spectrophotometry is typically used for evaluating the degree of turbidity [18]. As shown in Figure 1A, the transmittance of Cs (10–20 mg/mL) in hydrochloric acid solution was close to 90%, indicative of a high degree of transparency. In contrast, its light transmittance reduced sharply by 40–50% at concentrations of 20–25 mg/mL. This demonstrated that the amine groups on Cs formed a salt with the hydrogen ions in the acid, such that the original hydrogen bond of the Cs was destroyed and dissolved. Therefore, a hydrochloric acid solution with a Cs concentration of 10 mg/mL was used for freeze-drying. As shown in Figure 1B, right panel, uniform lyophilized Cs powder was obtained after freeze-drying. Compared with the regular Cs powder, which is almost insoluble in Met-containing physiological saline, the equivalent quantity of lyophilized Cs powder was completely dissolved (Figure 1C), indicating a notable increase in solubility following the freeze-drying procedure. Furthermore, the lyophilized Cs powder displayed a well arranged and finely networked structure, in contrast to the disordered structure of regular Cs powder (Figure 1D), demonstrating a notable improvement in the microstructure following freeze-drying. In addition, the Tyndall effect assay indicated that only MLCH showed a significant Tyndall effect (Figure 1E), demonstrating its colloidal characteristics. Subsequently, we used infrared spectra to determine whether Cs and metformin formed intermolecular chemical bonds or if Cs served as a carrier to load metformin. As shown in Figure 1F, the absorption peaks of Met at 3368 and 3292 cm^−1^ represent the asymmetric and symmetrical stretching vibration of -NH. The absorption peak at 3150 cm^−1^ is the stretching vibration absorption peak of -NH, and the absorption peak at 1621 cm^−1^ is the stretching vibration of C=N. The double absorption peaks at 1472 and 1446 cm^−1^ are the symmetric bending vibration absorption peaks of the methyl CH groups, indicating that there were no significant differences amongst the characteristic peaks between the infrared spectra of MLHC and Met. In addition, ^1^H NMR spectra proved that no new peaks were generated (Figure 1G). All these results showed that MET was wrapped in loose and porous Cs rather than formed intramolecular chemical bonds between MET and Cs (Figure 1H).

### 2.2. MLCH Exhibits Favorable Physical and Chemical Properties and Can Be Administered Intravesically

**Figure 2:**
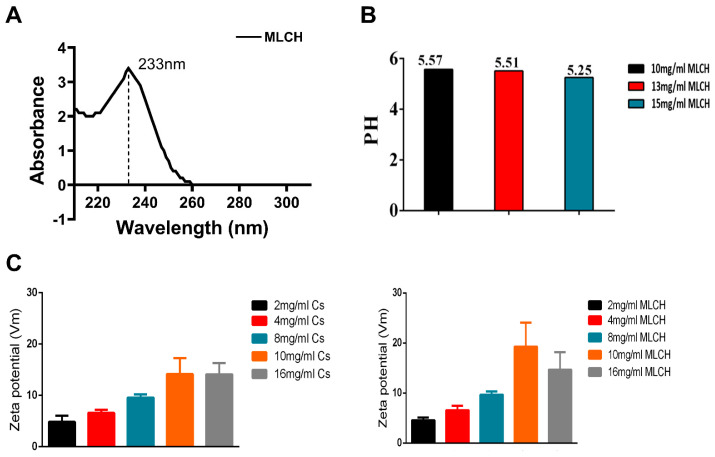
Characterization of MLCH. (**A**) The UV spectra of MLCH showed the characteristic absorption peak of Met at 233 nm. (**B**) After freeze-drying, MLCH had a pH of ~5, which satisfied the requirements for bladder perfusion. (**C**) MLCH had a positive ζ potential, which is beneficial for adhesion to the bladder mucosa. MLCH, metformin-loaded Cs hydrogel.

In order to obtain the optimal MLCH, the features of MLCH prepared with different concentrations of Cs were detected. The UV spectrum of MLCH displayed a characteristic absorption peak at 233 nm, indicating the presence of Met (Figure 2A). Moreover, the pH of MLCH was ~5, meeting the requirements for bladder perfusion (Figure 2B).

The ζ potentials of all MLCH solutions were positive due the presence of the positively charged carbohydrates of Cs (Figure 2C). Of note, the addition of Met increased the ζ potential value (Figure 2C, right panel) compared with the Cs hydrogel alone (Figure 2C; left panel), indicating a favorable physical interaction between Met and lyophilized Cs hydrogel. More importantly, a maximum ζ potential was obtained when 10 mg/mL of lyophilized hydrogel was used, demonstrating the optimum physical interaction between these two substances (Figure 2C). High ζ potentials favor the adherence of a drug to the negatively charged bladder membrane (Figure 2C). All these results indicated that 10 mg/mL of CS was the most suitable vector for metformin.

### 2.3. MLCH Shows a Robust Inhibitory Effect against BC Cell Growth In Vitro

**Figure 3:**
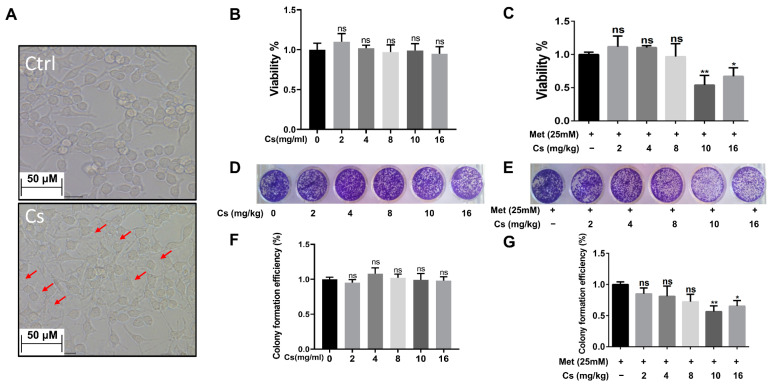
Different concentrations of Cs hydrogels and Met synergistically inhibited bladder cancer growth in vitro. (**A**) MB49 cells were treated with Cs hydrogel for 2 h. Then, the cells were cultured for 48 h after removal of the Cs hydrogels. Images were taken using an inverted microscope (Red arrows indicate that Cs shell adhered to the cells). (**B**,**C**) MB49 cells were treated with Cs or MLCH for 2 h. Then, the cells were cultured for 48 h after removal of the Cs or MLCH, the viability of MB49 cells was assessed by MTT assay. (**D**,**E**) MB49 cells were treated with Cs or MLCH for 2 h (twice in 1 week) and then cultured with a complete medium; the viability of MB49 cells was assessed by colony formation assays. (**F**,**G**) Quantification of the colony formation assays. The wells were scanned at a wavelength of 550 nm. Data are presented as the mean ± standard deviation of five independent experiments. * *p* < 0.05, ** *p* < 0.01, ns = not significant. MLCH, metformin-loaded chitosan hydrogel; Ctrl, control; Cs, chitosan alone; Met, metformin alone.

The next question was whether the MLCH prepared at 10 mg/mL of Cs had the best antitumor effect. Since MB49 is one of the most commonly used murine BC cell lines for the establishment of an orthotopic BC model in immune complete mice, the inhibitory effect of MLCH was assessed using this cell line. As shown in Figure 3A, after removing the culture medium, the Cs shell adhered to the cells. The Cs had no inhibitory effect on the growth of tumor cells, indicating that Cs had good adhesion and safety. The optimal ratio of MLCH was assessed using MTT assays. Cell viability was assessed via treatment with Cs hydrogel alone or with MLCH formulations of different Cs concentrations for 2 h. As shown in Figure 3B, the Cs hydrogels alone did not inhibit the growth of tumor cells, indicative of their low toxicity. In contrast, MLCH exhibited a potent inhibitory effect against cell viability. Moreover, there were significant differences in cells treated with MLCH via 10 mg/mL Cs hydrogels, MLCH via 16 mg/mL Cs hydrogels, and Met alone, at all detected amounts, in MB49 cells. Notably, MLCH in 10 mg/mL Cs hydrogel exhibited the best inhibitory effect (Figure 3C), and thus, this ratio of Cs hydrogel:Met was considered optimal. Furthermore, colony formation assays showed that MLCH in 10 mg/mL Cs hydrogels exhibited the optimum anticancer activity, in agreement with the MTT assay (Figure 3D–G). Taken together, these data provide solid evidence that 10 mg/mL Cs-prepared MLCH had the best anticancer activity.

### 2.4. Intravesical Treatment of MLCH Possesses Potent Anticancer Effects In Vivo

**Figure 4:**
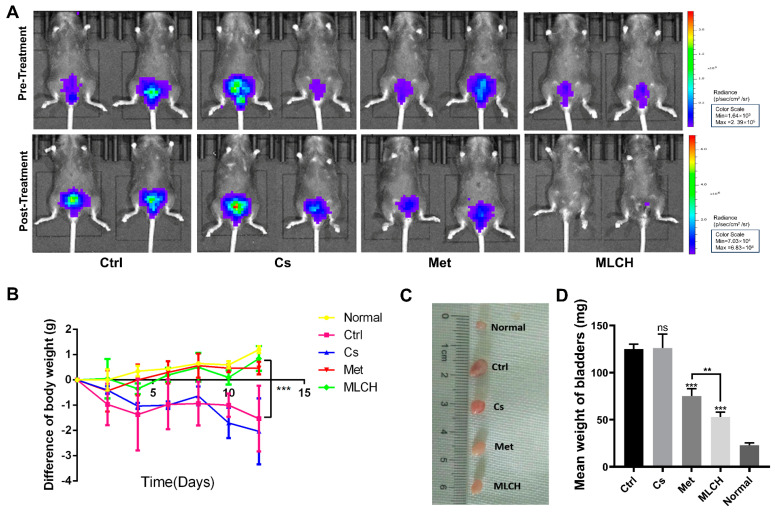
MLCH inhibits BC growth in vivo when administered intravesically. (**A**) Bioluminescent images of the mouse orthotopic implantation model following the different treatments. (**B**) Total weight of mice during the entire procedure. (**C**) Images of the explanted bladder tissues. (**D**) Weights of the mouse bladders, including those of mice that died before the end of the experiment. Data are presented as the mean ± standard deviation of five independent experiments. ** *p* < 0.01, *** *p* < 0.001, ns = not significant. BC, bladder cancer; MLCH, metformin-loaded chitosan hydrogel; Ctrl, control; Cs, chitosan alone; Met, metformin alone.

Orthotopic mouse tumor models are useful tools to assess the effects of intravesical localized treatments. The anti-tumor activity of MLCH was assessed in vivo using a well-established orthotopic tumor mouse model. Met alone exhibited significant inhibitory effects against bladder tumor growth compared with the control mice. MLCH demonstrated a more potent inhibitory effect against bladder tumor growth compared with Met alone, demonstrating the beneficial effects of the novel formulation for BC treatment in vivo. No obvious inhibitory effects against bladder tumor growth were observed in the Cs group (Figure 4A). During the course of the animal experiments, the total body weight of the animals was monitored to assess whether any of the treatments exerted any toxic effects. Consistent with the in vitro experiments, mice with orthotopic tumors that were not treated with Met or MLHC exhibited significant decreases in body weight due to the tumor and they were immediately euthanized when the maximum weight loss of the mice exceeded 20%. The decrease in body weight caused by BC was alleviated in the Met group. Consistent with the results of the MTT assay, in the Cs group, the body weight decreased in a manner similar to that observed in the control group; thus, Cs hydrogel alone did not exert any beneficial effects. Intravesical administration of MLCH and Met resulted in significant alleviation of the decrease in body weight (Figure 4B). In addition, as shown in Figure 4C,D, the heaviest bladders were measured in the control and Cs groups due to the formation of the tumors. In contrast, the bladder weights in both the Met and MLCH groups were significantly lower compared with the control and Cs groups and the reduction was greater in the MLCH group.

Taken together, MLCH displayed excellent antitumor effects, as demonstrated by the inhibition of growth of orthotopic tumors, improvement in whole body weight, and attenuation of the tumor-induced increase in bladder weight.

### 2.5. MLCH Increases Phosphorylation of AMPKα in BC Tissues

**Figure 5:**
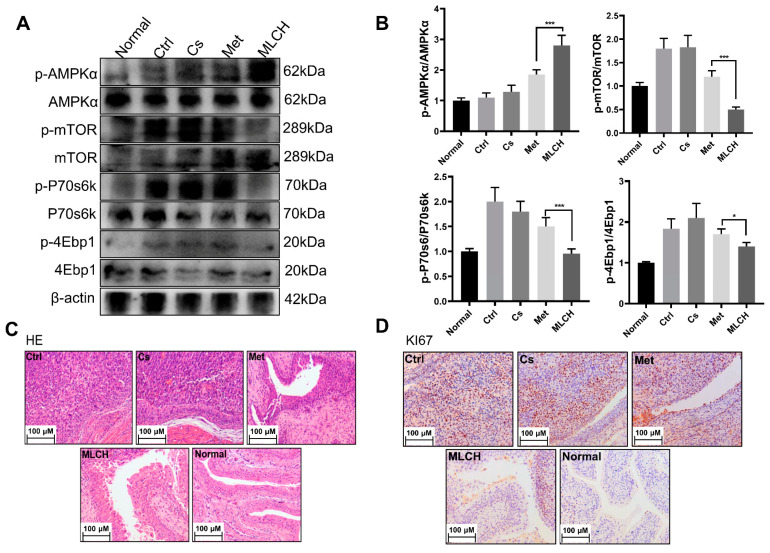
Inhibitory effect of MLCH on AMPKα signaling pathways in bladder cancer. (**A**) Western blot analysis of p-AMPKα, p-mTOR, p-P70s6k, and p-4Ebp1 protein expression in bladder tissues from the mice in the five different groups. (**B**) Relative levels of p-AMPKα, mTOR, P70s6k, and 4Ebp1 are shown as the mean ± the standard error of the mean. * *p* < 0.05, *** *p* < 0.001. (**C**,**D**) Histological sections of the bladder tissues were subjected to hematoxylin and eosin staining or were used for immunohistochemistry analysis of Ki67 expression to confirm the presence or absence of tumors. MLCH, metformin-loaded chitosan hydrogel; Ctrl, control; Cs, chitosan alone; Met, metformin alone; p-, phosphor; P70s6k, ribosomal protein S6 kinase; 4Ebp1, eukaryotic translation initiation factor binding protein 1.

It is well established that the anticancer activity of Met is mediated by its ability to activate the AMPKα pathway [19,20]. By examining the levels of p-AMPKα and its downstream proteins, including p-mTOR, p-P70s6k, and p-4Ebp1, it was found that MLCH significantly increased the phosphorylation of AMPKα and reduced the phosphorylation of the mTOR signaling pathway-related proteins induced by Met (Figure 5A,B). This exploration of molecular mechanisms confirmed that MLCH killed BC cells via the same pathway as Met; Cs hydrogel notably amplified the effects of Met by acting as an adjuvant. Furthermore, compared with the Met treatment group, tumor growth in the MLCH treatment group was significantly lower than in the Met group (Figure 5C). Moreover, the staining results of Ki67 as a tumor marker demonstrated similar efficiency patterns (Figure 5D). Thus, the intravesical administration of MLCH exhibited more potent anti-BC ability and its effects were mediated by regulation of the AMPKα pathways.

### 2.6. MLCH Exhibits No Detectable Toxic Effects and Remains Localized in the Bladder

**Figure 6:**
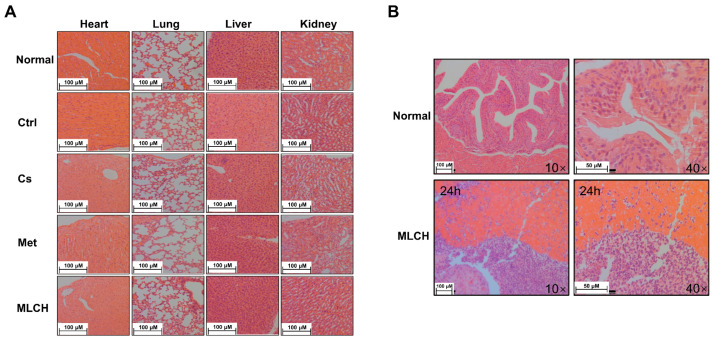
Pathological examination of the vital organs. (**A**) Pathological examination of the vital organs in the five groups of mice. Hematoxylin and eosin staining were used for pathological examination of tissue sections of the heart, lung, liver, and kidneys. (**B**) Observation of the inner wall of the bladder showed Cs was retained for ≥24 h. The bladder tissue was assessed using hematoxylin and eosin staining following the administration of MLCH for 24 h to observe the retention of Cs. MLCH, metformin-loaded chitosan hydrogel; Ctrl, control; Cs, chitosan alone; Met, metformin alone.

To examine whether MLCH induced any toxic effects, tissues from the heart, lungs, liver, and kidneys of mice were taken for pathological examination after two weeks of bladder perfusion. Interestingly, no significant differences between the respective tissues were observed in the five groups (Figure 6A). This indicated that the effects of intravesical administration of MLHC in the bladder were limited to the bladder and it had no toxic effects on the other organs. Thus, this administration route did not induce any observable side effects.

Moreover, to detect the presence of MLCH in the mouse bladder, the inner wall of the bladder 24 h after Cs administration was examined. As shown in Figure 6B, compared with the normal bladder tissues, the presence of Cs hydrogels was observed in the MLCH-treated group, indicating that the location of Cs hydrogels was restricted to the bladder wall.

## 3. Discussion

Cs has received a significant amount of attention in the development of in situ local treatments for tumor therapy, particularly as a potential delivery vehicle in oral and ophthalmic delivery systems [13,21,22,23]. Hydrogels, cross-linked polymers with high water content, can be used to continuously deliver various therapeutic agents locally [24,25,26,27]. In addition, Cs structures with a positive charge adhere electrostatically to the mucosal wall of the bladder, which possesses an overall negative charge; thus, it was used to develop a novel formulation of Met [28,29]. By combining their respective advantages, a novel MLCH formulation was constructed for controlled topical use.

Cs has been approved for application in the medical field as its safety profile has been strictly evaluated and is well established [30,31,32]. Moreover, its superior physical features are important for preparing excellent formulations of various drugs. In the present study, no chemical modifications were made to Cs when preparing the lyophilized powder using the freeze-drying method. There were no significant differences between the characteristic peaks between the infrared spectra of MLCH and Met, indicating that the new formulation did not contain any new compounds. Moreover, the UV spectrum of MLCH showed a characteristic absorption peak of Met at 233 nm. Therefore, this formulation does not require any additional safety assessments for future clinical applications. In addition, the freeze-drying method not only effectively removed the volatile acid of Cs but also directly adjusted the pH to ~5, thereby avoiding the risk of irritation of the mucosa under acidic conditions and further promoting patient compliance.

The unique barrier structure of the bladder results in only a small amount of accumulation of a drug at the effective site of the bladder when a drug is administered orally, underlying the low efficiency of drugs used for the treatment of bladder diseases when administered systemically. Although intravesical administration can reduce the intact dose of drugs required by delivering drugs directly into the bladder, thus reducing the side effects of higher doses [33], the urine regularly emptied from the bladder significantly reduces the retention time of the drug in this organ and, therefore, frequent administration is required. Drug-loaded Cs adheres to the bladder wall and prolongs the action time of drugs. The prepared Cs lyophilized powder showed a loose and porous three- dimensional network structure with high solubility. The drug effectively prepared under these conditions had a certain viscosity. At the same time, the ζ potential of the new Met dosage form was positive, particularly when the Cs concentration was 10 mg/mL. MLCH had the maximum potential value and this allowed the drug to adhere to the negatively charged bladder membrane. The high concentration of Cs effectively delayed the release of the drug over a 24-h period. Thus, it is speculated that the novel Cs formulation exhibited superior structural features and may prolong the life of the external solution medium entering the bladder wall, leading to a slower release of Met from the carrier matrix into the bladder wall. Furthermore, MLCH with optimized viscosity allowed for an extended duration of the action of Met and thus improved its therapeutic effects. To demonstrate the anti-tumor activity of this novel formulation of Met, comprehensive in vitro and in vivo assays were performed using cultured cells and advanced orthotopic tumor mouse models. The ideal formulation of MLCH, with suitable viscosity and turbidity, was obtained after optimizing the proper ratio of Cs hydrogel to Met. In vivo assays demonstrated the extended retention time and strong anti-tumor activity of this novel formulation. At the same time, Cs alone did not exhibit any significant anti-tumor properties. More importantly, histological analysis showed the excellent non-toxic properties of this novel preparation. Met is a strong AMPKα activator [34]. The novel formulation showed more potent functional activation of the AMPKα/mTOR pathway compared to Met alone.

**Figure 7:**
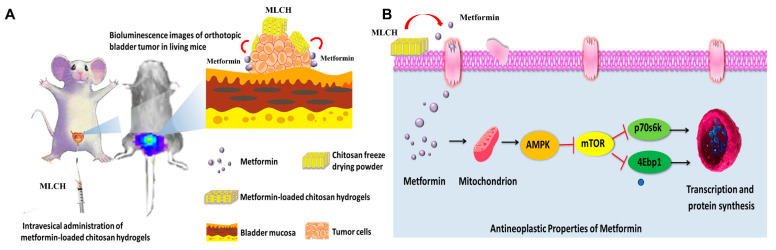
Scheme of the MLCH delivery system and mechanism of action. (**A**) MLCH exerts a beneficial therapeutic effect in an orthotopic mouse model of bladder cancer via extending the metformin retention time. (**B**) MLCH activates AMPKα and down-regulates the mTOR signal pathway, thereby inhibiting P70s6k and 4Ebp1 phosphorylation to block the tumor cell growth.

These findings may have been related to the fact that Cs remained in the bladder for a longer period of time. Cs, as a drug carrier, prolongs the duration of Met, thereby improving the longevity of the effects of Met. However, in spite of the fact that MLCH could receive encouraging therapeutic effects via intravesical administration, nevertheless, how to deliver metformin targeted to other tumors (such as lung cancer, liver cancer, etc.) so as to achieve an anti-tumor concentration remains a research orientation seriously worth exploring. In addition, studies in large animal models are required to establish whether MLCH would be clinically acceptable. Nevertheless, despite several possible shortcomings, MLCH significantly enhanced the antitumor effects of metformin in BC (Figure 7) and it represents a safe and highly efficient potential approach for convenient treatment of BC via intravesical administration, offering bright prospects for improving the treatment of BC.

## 4. Materials and Methods

### 4.1. Reagents

Met (chemical name, 1,1-Dimethylbiguanide hydrochloride) was purchased from Sangon Biotech Co., Ltd. (Shanghai, China). Cs (85% degree of deacetylation) was purchased from Haidebei Marine Biological Engineering Co., Ltd. (Jinan, China). D-Luciferin was purchased from Yanjian Biotechnology Co., Ltd. (Yanji, China).

### 4.2. Preparation of Freeze-Dried Cs Powder

A pre-determined weight of regular Cs was dissolved in 1% hydrochloric acid (*v*/*v*). The suspension was centrifuged at 30,000 rpm at 4 °C for 10 min using an ultracentrifuge to remove the insoluble particles in the Cs solution. The obtained transparent hydrogel was laid flat and frozen at −80 °C for pre-freezing and the freeze-drying temperature and vacuum degree were adjusted to obtain a lyophilized powder of Cs. Finally, the lyophilized powder was mixed with Met-containing physiological saline of different ratios to obtain MLCH of various viscosities.

### 4.3. Physicochemical Characterization

Turbidity was measured via UV spectrophotometry (Beckman Coulter, Inc., Brea, CA, USA); 1% (*v*/*v*) hydrochloric acid solution served as a reference. Using a wavelength range of 200–800 nm, the absorbance of hydrogels with 10, 15, 20, 25, and 30 mg/mL Cs was measured. Turbidity was estimated by calculating the transmittance at this wavelength from the absorbance (A) value at 600 nm.

The viscosity of the hydrogels was measured using an Ubbelohde viscometer (Baoshan Qihang Glass Instrument Factory, Shenzhen, China). Briefly, 10 mL of polymer hydrogel was injected into the viscometer, maintaining the liquid at a height below atmospheric pressure. When the air pressure in the viscometer was adjusted to maintain atmospheric pressure, the solution dripped due to gravity. The duration of time in sec between the liquid level passing through the upper and lower graduation marks was measured. The measurements were repeated three times and the difference between the measurements did not exceed 0.1 s. The outflow time (T) of the test hydrogel was the mean of three measurements. The kinematic viscosity calculation formula used was viscosity = viscosity capillary inner diameter × viscometer constant × T.

Tyndall effect assays were performed by preparing equivalent volumes of MLCH and Met solution in two test tubes. The light from a laser pointer was illuminated with a white background. The images were taken vertically for recording.

Fourier transform infrared spectroscopy was performed using a Fourier transform infrared spectrometer (Spectrum One B; Perkin Elmer, Inc., Waltham, MA, USA); the scanning range was 4000–500 cm^−1^.

The UV absorption spectra of MLCH of different concentrations were measured. MLCH was placed in a dialysis bag and dialyzed in the release media of PBS at 37 °C. Then, a 1 mL hydrogel solution of release media was collected. The amount of drug released was measured using a UV spectrometer (Beckman Coulter, Inc., Brea, CA, USA).

ζ potential analysis was performed using a Zetasizer according to the manufacturer’s protocol (Malvern Nano series, Malvern, UK). Briefly, a diluted suspension of MLCH was prepared in PBS to measure the ζ potential. All analyses were performed in triplicate.

### 4.4. Cell Viability Assay

The murine BC cell line, MB49, was kindly provided by Dr P Guo (Institute of Urology, Xi’an Jiaotong University). MB49 cells were cultured in DMEM (Hyclone; Cytiva) supplemented with 10% FBS (Hyclone; Cytiva) and 1% penicillin/streptomycin. Cells were cultured at 37 °C with 5% CO_2_ in a humidified incubator and were frequently assessed for mycoplasma contamination.

Cell viability was assessed using an MTT assay using a microplate reader (SYNERGY HTX; BioTek Instruments, Inc., Winooski, VT, USA). Briefly, cells were seeded at a density of 8 × 10^3^ cells/well in 96-well culture plates and incubated in a medium supplemented with 10% FBS. After 12 h, cells were treated with different concentrations of MLCH or with Met alone for 2 h. Then, cells were cultured for a further 48 h in the culture medium. The tetrazolium salt of MTT (50 μL; Sigma-Aldrich; Merck KGaA, St. Louis, MO, USA) was dissolved in Hank’s balanced salt solution to a concentration of 2 mg/mL, added to each well, and incubated for 5 h. Subsequently, the medium was aspirated from each well and 150 μL DMSO (Sigma-Aldrich; Merck KGaA) was added to dissolve the formazan crystals. The absorbance was measured using a microplate reader at 490 nm against the reference absorbance at 630 nm. Data were statistically analyzed using SPSS version 16.0 (SPSS, Inc., Chicago, IL, USA).

### 4.5. Clonogenic Assay

MB49 BC cells (8 × 10^3^ cells/well) were plated in a 24-well plate and incubated for 24 h at 37 °C. Subsequently, cells were treated with Met alone or with different concentrations of MLCH for 2 h in media supplemented with 10% FBS. Then, the drug-containing medium was replaced with DMEM. The cells were then treated twice with the different preparations for 2 h each time, fixed with 10% formaldehyde after 7 days of culture, and stained with 0.1% crystal violet at room temperature for 4 h. Absorbance was measured using a microplate reader at a wavelength of 550 nm.

### 4.6. Orthotopic Implantation and Intravesical Treatment

Female C57BL/6 mice were purchased from Hunan SJA Laboratory Animal, Co., Ltd. (Changsha, China). Animals were housed in a specific pathogen-free animal facility.

MB49 cells (transfected with luciferase) in the exponential growth phase were harvested and the cell density in the collection tubes was counted using a cell counter. A total of 1.2 × 10^5^ MB49 cells in 0.1 mL PBS were injected into the bladder walls of mice using 1 mL syringes following catheter scratching to establish the orthotopic BC mouse model, as described previously [35]. The mice were divided into five groups: (i) normal female C57BL/6 mice administered with 50 μL saline intravesically but not injected with tumor cells and female C57BL/6 mice with orthotopic BC treated with (ii) 50 μL saline (Ctrl); (iii) Cs hydrogel (10 mg/mL; Cs); (iv) Met (80 mM; Met); or (v) 10 mg/mL Cs hydrogel loaded with 80 mM Met (MLCH). Each group consisted of six female C57BL/6 mice. All treatments began on the second day after the successful establishment of the orthotopic model, were administered once a week, and lasted for 2 weeks. Mice were anesthetized via intraperitoneal injection of sodium pentobarbital (100 mg/kg) and then sacrificed via cervical dislocation. The tumor burden was assessed using a Xenogen In Vivo Imaging system (PerkinElmer, Inc., Waltham, MA, USA).

### 4.7. Histological Analysis

At the end of the study, the animals were sacrificed and the bladder, heart, lung, liver, and kidneys were removed. Portions of organ tissues were used for protein extraction or immediately immersed in 4% neutral buffered formalin for histological slide preparations. The samples used for histological analysis were subsequently processed using conventional histological techniques. Hematoxylin and eosin, as well as Ki67 staining of 7 μm tissue sections, were performed. The slides were observed using a light microscope (DFC450C; Leica Microsystems GmbH, Wetzlar, Germany). Pathological evaluation of tissues was performed to confirm the presence of tumors, as well as other pathological features.

### 4.8. Western Blotting

Total tissue protein was extracted using a grinder (Wuhan Servicebio Technology Co., Ltd., Wuhan, China) and ~10 μL protein tissue fluid containing 30 μg total protein was resolved using SDS-PAGE and transferred to PVDF membranes, after which the membranes were cut according to the corresponding molecular weight of the protein. Membranes were incubated with the primary antibody (Cell Signaling Technology, Inc., Danvers, MA, USA) and incubated overnight at 4 °C. The following day, membranes were washed with PBS containing 1% Tween−20, blotted with the secondary antibody for 1 h at room temperature, and then washed again three times. Pierce Super Signal chemiluminescent substrate (Thermo Fisher Scientific, Inc., Waltham, MA, USA) was used to visualize the signals and the blot was imaged immediately using a ChemiDoc system (Tanon 4600; Tanon Science and Technology Co., Ltd., Shanghai, China). Densitometry analysis was performed using ImageJ 1.8.0 software (National Institutes of Health, Bethesda, MD, USA).

### 4.9. Statistical Analysis

Data from the in vitro experiments are presented as the mean ± standard deviation. In vivo experimental results are expressed as the mean ± the standard error of the mean. Differences between groups were compared using a Student’s *t*-test (two groups) or a one-way ANOVA with a post hoc least significant difference test (more than two groups). Statistical analysis was performed using GraphPad Prism version 6.0 (GraphPad Software, Inc., La Jolla, CA, USA) or Origin version 8.0 (OriginLab Corporation, Northampton, MA, USA). *p* < 0.05 was considered to indicate a statistically significant difference.

## Data Availability

The authors declare that the data supporting the findings of this study are available within the paper or from the corresponding authors upon request.

## Supplementary Materials

The following supporting information can be downloaded at: https://www.mdpi.com/article/10.3390/molecules28186720/s1. Figure S1: The structure of metformin.
