# Supplementary material for: Metformin-Loaded Chitosan Hydrogels Suppress Bladder Tumor Growth in an Orthotopic Mouse Model via Intravesical Administration
Source: Molecules. 2023 Sep 20;28(18):6720. doi: 10.3390/molecules28186720 (PMC10534355; doi:10.3390/molecules28186720)
Supplement: Supplementary file 1 [file molecules-28-06720-s001.zip › molecules-2614574-supplementary.pdf]

Supplementary information

Metformin-Loaded Chitosan Hydrogels Suppress Bladder Tumour Growth in an Orthotopic Mouse Model via Intravesical Administration

Xingjian Zhang[1,†], Xin Hu[1,†], Yijun Xie[1], Lejing Xie[1], Xiangyi Chen[1], Mei Peng[1], Duo Li[1], Jun Deng[1], Di Xiao[1]* and Xiaoping Yang [1,2]*

[1]Key Laboratory of Study and Discovery of Small Targeted Molecules of Hunan Province, Key Laboratory of Chemical Biology & Traditional Chinese Medicine Research of Ministry of Education, Department of Pharmacy, School of Medicine, Hunan Normal University, Changsha, Hunan, China.
[2]Key Laboratory of Protein Chemistry and Developmental Biology of Fish of Ministry of Education, Hunan Normal University, Changsha, Hunan, China.
† These authors contributed equally to this work.

* Correspondence: dixiao@ hunnu.edu.cn; (D.X.); xiaoping.yang@hunnu.edu.cn (X.Y.); Tel/Fax: 86-158-7406-6132(X.Y.).

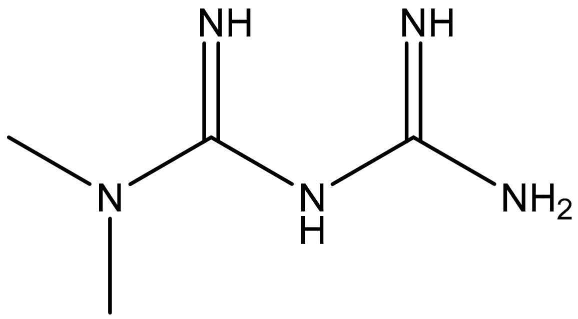

Figure S1. The structure of metformin.
